# Milk and Its Products

**Published:** 1887-08

**Authors:** 


### MILK AND ITS PRODUCTS.

On the 4th of July, a large number of families in the lower portion of New York City, were seriously poisoned by eating ice cream, the product of a single manufacturer.

The investigation of the subject was turned over to the Board of Health, and a microscopic analysis revealed the presence of *green cells* in the objectionable compound, which are a pernicious fungus, liable to develope in *pure milk* under certain conditions, which it is almost impossible to guard against.

In the present instance, the maker of the poisonous cream was doubtless innocent of any offensive or injurious adulteration of it, and the poisoning is attributed to natural causes, which could not have been anticipated or foreseen by the exercise of ordinary diligence.

In view of these facts, the following article upon cows' milk, contributed to the *Medical Summary*, will be read with interest:

"Milk, which more than any other article of food, is consumed by a greater number of the population, from the new born babe to the adult up to the age of four-score and ten, in every sphere in life. The necessity of care in the feeding of animals, and the care of the product of the cow after its passage into the hands of the dairyman and thence to the consumer cannot, therefore, be too strenuously insisted upon, and every restraint upon its distribution in the purest state, should be insisted upon by legal restraint, by the medical profession and by the public at large, and the severest punishment meted out against the carelessness and adulterations of dealers in the article."

We allude to the subject to notice a remarkable out-break of scarlatina in certain streets of London, probably a year ago, which was directly traced to milk furnished from a single farm. In a similar investigation a few years ago, a contagious fever was shown to have been originated in a dairy farm where the milkpans were washed with water taken from a contaminated cistern. The disease was found only in those houses on certain streets, which were supplied with milk from this farm. In the more recent instances, scarlatina has been traced by Dr. Klein, to one or two cows which were found to have diseased udders. The milk was affected with the germs of disease in the process of milking, and then became a vehicle for spreading it from house to house. Dr. Klein succeeded in producing the disease artificially by inoculating four calves with matter taken from the cows. The calves manifested all the characteristic symptoms of human scarlatina.

Milk has been shown to be not only a disease carrier, but also a germ multiplier. The sores on the cows were not considered serious at the dairy farm, yet minute particles from them, communicated the disease to a large number of families. This seems to be a conclusive proof that milk is a good medium for the multiplication of the germs of a contagious disease.

A case was also reported of milk poisoning, a year or two ago, at a hotel at one of the well-known watering places along the New Jersey coast, where about forty persons were taken violently sick. Doctors were summoned, and, after prompt and vigorous treatment, all were declared out of danger except one, the daughter of the proprietor of the hotel. She was confined to her bed for several days. The milk was supplied by a dealer, who supplied another hotel where there was similar trouble about ten days before."

It is a well understood fact, that milk is largely supplied in nearly all thickly populated cities, from stall fed cows, which, for the most part, are anything but properly fed and neatly housed. This is particularly the case in New York, Brooklyn and Jersey City. A few years ago some disgusting developments were made, growing out of this custom. Cows, to be healthy, require the open range of green fields and well watered pastures. Unhealthy cows are almost sure to impart disease by the free use of their milk, which is likely to be tainted with the poisons which enter into the air they breathe and the food upon which they are kept.

Dr. Novy, of Ann Arbor, says that the poison in ice cream, is invariably due to the presence of decomposition products, formed from the nitrogenous constituents of the substance, and as many of the substances have been obtained from putrefying bodies, they are also sometimes known as "animal or cadavar alkaloids," some of which are violent poisons, while others are harmless.

The demand for cheap ice cream has become so enormous, as to lead many dealers into the use of animal gelatine in its manufacture. They are certainly not conscious of the fact that much of the gelatine in the market is but imperfectly cured, and therefore, liable to decompose. Gelatine furnishes a magnificent soil for the growth of bacteria—in fact, it is used as a culture soil for these germs by bacteriologists. For these considerations it should not be used in the manufacture of ice cream. Far better would it be to follow these simple and harmless directions :

First—The milk taken from the cow should be immediately cooled and kept cool.

Second—The cream should be gathered from perfectly fresh, cold milk.

Third—The custard should be frozen as soon as made, or else be kept in a refrigerator.

